# Neutrophil-to-lymphocyte ratio predicts prostatic carcinoma in men undergoing needle biopsy

**DOI:** 10.18632/oncotarget.5081

**Published:** 2015-08-20

**Authors:** Takashi Kawahara, Sachi Fukui, Kentaro Sakamaki, Yusuke Ito, Hiroki Ito, Naohito Kobayashi, Koji Izumi, Yumiko Yokomizo, Yasuhide Miyoshi, Kazuhide Makiyama, Noboru Nakaigawa, Takeharu Yamanaka, Masahiro Yao, Hiroshi Miyamoto, Hiroji Uemura

**Affiliations:** ^1^ Department of Urology, Yokohama City University, Graduate School of Medicine, Yokohama, Japan; ^2^ Departments of Urology and Renal Transportation, Yokohama City University Medical Center, Yokohama, Japan; ^3^ Department of Biostatistics, Yokohama City University Graduate School of Medicine, Yokohama, Japan; ^4^ Departments of Pathology and Urology, Johns Hopkins University School of Medicine, Baltimore, USA

**Keywords:** Clinical Section, prostate cancer, biomarker, neutrophil-to-lymphocyte ratio, prostate needle biopsy

## Abstract

Neutrophil-to-lymphocyte ratio (NLR), a simple marker of systemic inflammatory response, has been demonstrated as an independent prognosticator for some solid malignancies, including prostate cancer. In the present study, we evaluated the role of NLR in men who underwent prostate needle biopsy for their initial diagnosis of prostatic carcinoma. Both complete blood counts and free/total (F/T) prostate-specific antigen (PSA) ratio were examined in a total of 3,011 men in our institution. Of these, 1,207 had a PSA level between 4 and 10 ng/mL, and 357 of 810 who subsequently underwent prostate needle biopsy were found to have prostatic adenocarcinoma. NLR value was significantly higher in men with PSA of ≥ 20 ng/mL than in those with PSA of < 20 ng/mL (*p* < 0.001). NLR was also significantly higher in men with positive biopsy than in those with negative biopsy (*p* < 0.001). Using NLR cut-off point of 2.40 determined by the AUROC curve, positive/negative predictive values of NLR alone and NLR combined with F/T PSA ratio (cut-off: 0.15) were 56.6%/60.8% and 80.7%/60.1%, respectively. Multivariate analysis revealed that not only F/T PSA ratio (*HR* = 3.13) but also NLR (*HR* = 2.21) was an independent risk factor for prostate cancer. NLR is thus likely elevated in patients with prostate cancer. Accordingly, NLR, with or without combination with F/T PSA ratio, may function as a new biomarker to predict prostate cancer in men undergoing prostate needle biopsy.

## INTRODUCTION

Prostate-specific antigen (PSA), also known as human kallikrein 3, has been widely used for early detection of prostate cancer as well as monitoring of its treatment. However, nonmalignant conditions, especially benign prostate hyperplasia and acute prostatitis, often raise serum PSA, which complicates the diagnosis of prostate cancer using PSA measurement alone [1, 2].

Since its identification, various retrospective and prospective studies have assessed the usefulness of free/total (F/T) PSA ratio (cut-off points of 0.1–0.2) for differentiating between benign conditions and prostate cancer, especially in “gray-zone” patients who have PSA levels of 4–10 ng/ml [2–4]. Attempts have also been made to identify biomarkers that predict prostate cancer and its prognosis, but these often require quantitative PCR or immunohistochemical analysis. Therefore, additional biomarkers for prostate cancer whose values can be determined more simply and less expensively are needed.

Neutrophil-to-lymphocyte ratio (NLR) can be easily calculated from routine complete blood counts (CBCs) in peripheral blood. NLR has been suggested as not only a predictor of systemic inflammatory response in critical care patients but also a prognosticator for some solid malignancies including prostate cancer [5–14]. Men with elevated serum PSA yet without the diagnosis of prostate cancer were found to have a higher NLR compared with those with a normal PSA level [15], suggesting that certain markers of systemic inflammation and/or immune system activation were associated with an elevated serum PSA. The roles of NLR in monitoring of advanced prostate cancer such as castration-resistant tumors or that in patients who received docetaxel-based chemotherapy [15– 17], but not in initial diagnosis of prostate cancer, have been investigated. In the present study, we aim to assess the usefulness of NLR as a biomarker in men undergoing prostate needle biopsy. To our knowledge, this is the first study to evaluate NLR as a predictor of prostate cancer diagnosis.

## MATERIALS and METHODS

### Patients

**Figure 1:**
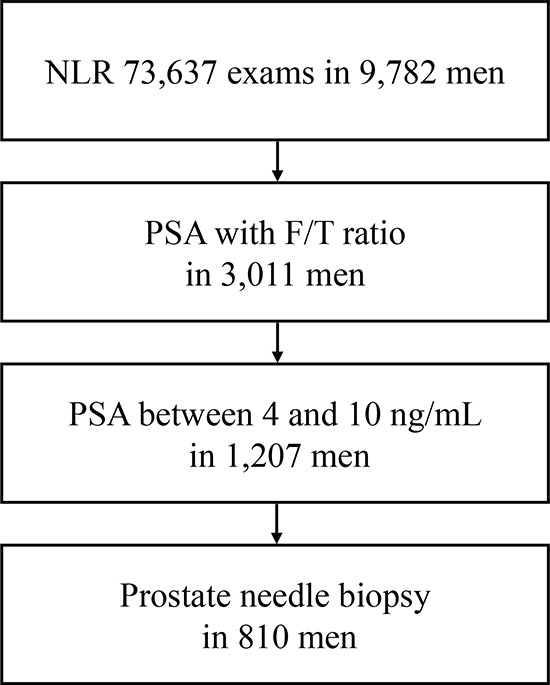
Patients' selection

A total of 73,637 CBC exams including absolute neutrophil and lymphocyte counts were performed in 9,782 men at the Department of Urology, Yokohama City University Hospital (Yokohama, Japan) from 1999 to 2015. Of these, 3,011 men had both CBCs and F/T PSA ratio. We further investigated 810 men who underwent prostate needle biopsy while showing PSA levels between 4 and 10 ng/mL [Fig 1]. Patients' age, NLR, PSA, and F/T PSA ratio are summarized in Table 1. The institutional review board of Yokohama City University approved this study.

**Table 1:** Patients' characteristics

Variables	median (range, mean ± SD)
Age (years)	73 (38–94, 73.4 ± 8.1)
NLR	1.94 (0.51–9.80, 2,14 ± 1.05)
PSA (ng/mL)	6.33 (4.0–10.0, 6,58 ± 1.60)
F/T PSA ratio	0.18 (0.04–0.74, 0.18 ± 0.07)

### Clinical and laboratory assessments

NLR was calculated using neutrophil and lymphocyte counts via CBCs obtained simultaneously with PSA and its F/T ratio. Prostate needle biopsy was typically performed within 4 weeks of blood tests. In some cases, prostate needle biopsy was not performed due to, for instance, the presence of urinary tract infection such as acute prostatitis, unsuspicious findings in pre-biopsy MRI, or advanced ages. We determined the cut-off point of NLR according to the sensitivity and specificity levels derived from area under receiver operator characteristics (AUROC) curve plotted using the presence or absence of prostatic carcinoma.

### Statistical analyses

Patients' characteristics and preoperative factors were analyzed using Mann-Whitney *U* test and chi-square test, using Graph Pad Prism (Graph Pad Software, La Jolla, CA, USA). Statistical significance was determined as *p* < 0.05.

## RESULTS

### NLR values and PSA

**Figure 2:**
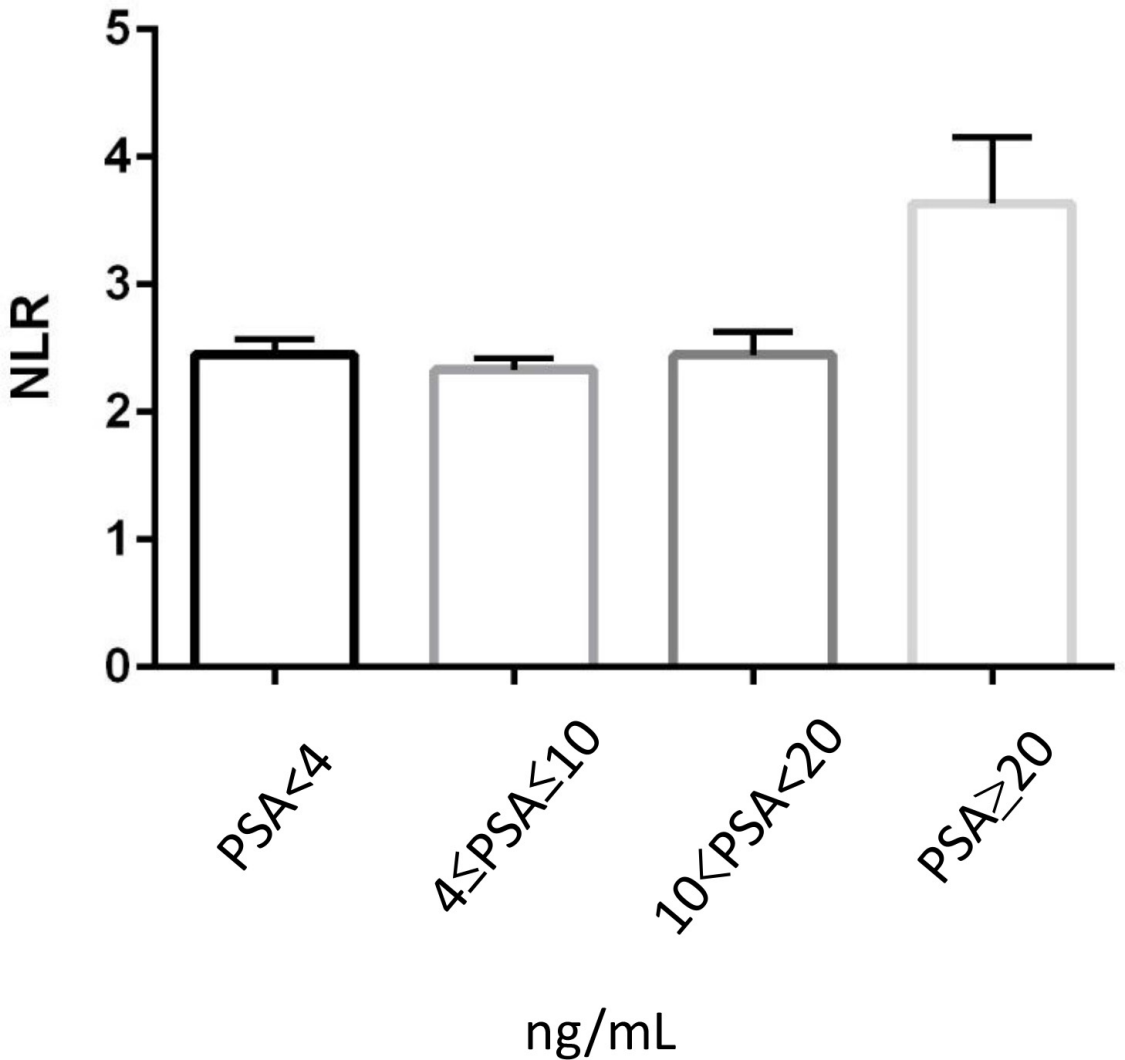
Correlation between PSA and NLR

There was no statistically significant difference (*p* = 0.242) in NLR value among men with PSA of < 4 ng/mL (median/mean ± SD: 2.05/2.45 ± 2.04), PSA between 4 and 10 (2.00/2.33 ± 1.63), and PSA of > 10 but < 20 (2.11/2.45 ± 1.84) [Fig 2]. Nonetheless, NLR was significantly (*p* < 0.001) higher in men whose PSA was > 20 (2.52/3.63 ± 4.20), compared with those with PSA of ≤ 20 (2.03/2.44 ± 2.05). NLR values were next assessed in men with a PSA level of 4–20. For this analysis, we first used the institutional cut-off point (0.15) of F/T PSA ratio in our clinics. NLR was found to be significantly (*p* < 0.001) higher in those with F/T PSA ratio of <0.15 (median/mean ± SD: 2.03/2.39 ± 1.78) than in those with F/T PSA ratio of ≥ 0.15 (1.90/2.21 ± 1.28).

### NLR values in men with vs. without prostate cancer

**Figure 3:**
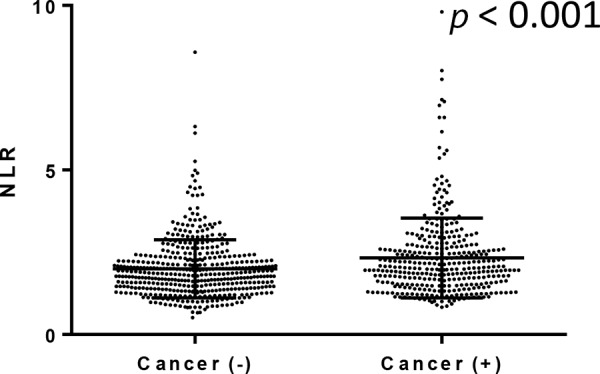
NLR in men undergoing prostate biopsy

**Figure 4:**
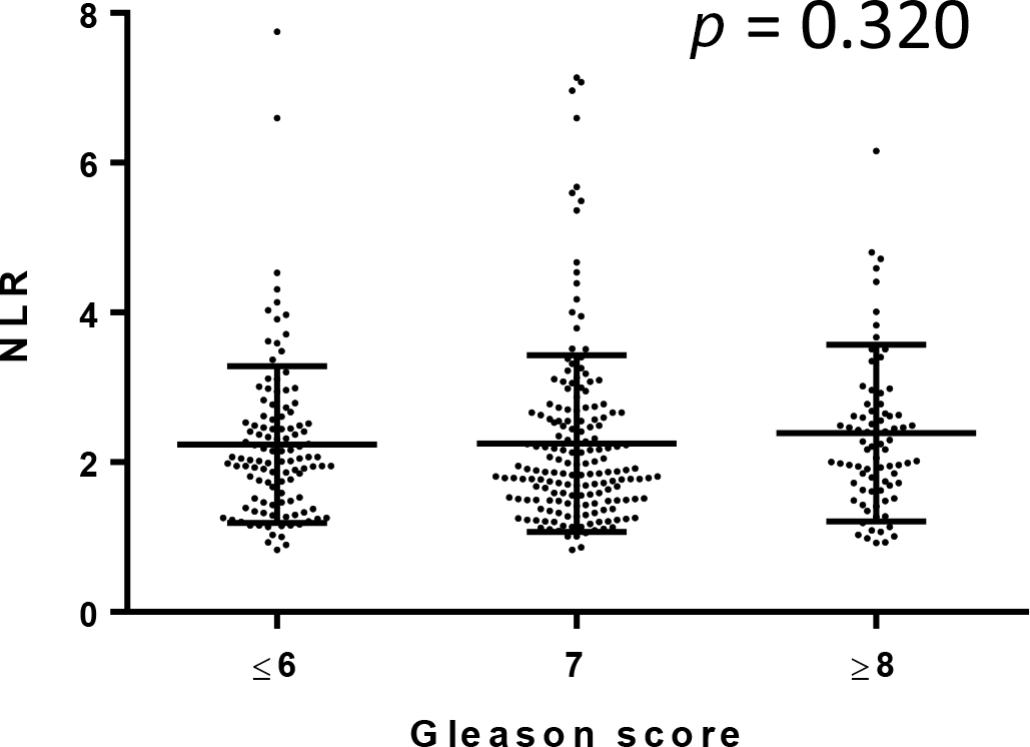
Correlation between NLR and Gleason score

We further compared NLRs in 810 men undergoing prostate biopsy. Of these, 357 (44.1%) were positive for prostatic carcinoma. None of these patients were found to have metastatic disease at the time of the biopsy. Gleason score (GS) was available in 344 of 357 patients: 5 (1.4%) GS5, 105 (30.5%) GS6, 163 (47.4%) GS7, 45 (13.1%) GS8, 21 (6.1%) GS9, and 5 (1.4%) GS10. NLR was again significantly (*p* < 0.001) higher in men in whom prostate cancer was found (median/mean ± SD: 2.02/2.33 ± 1.21) than in those without prostate cancer (1.84/2.00 ± 0.88) [Fig 3]. However, there were no significant correlations between NLR value and GS [Fig 4].

**Figure 5:**
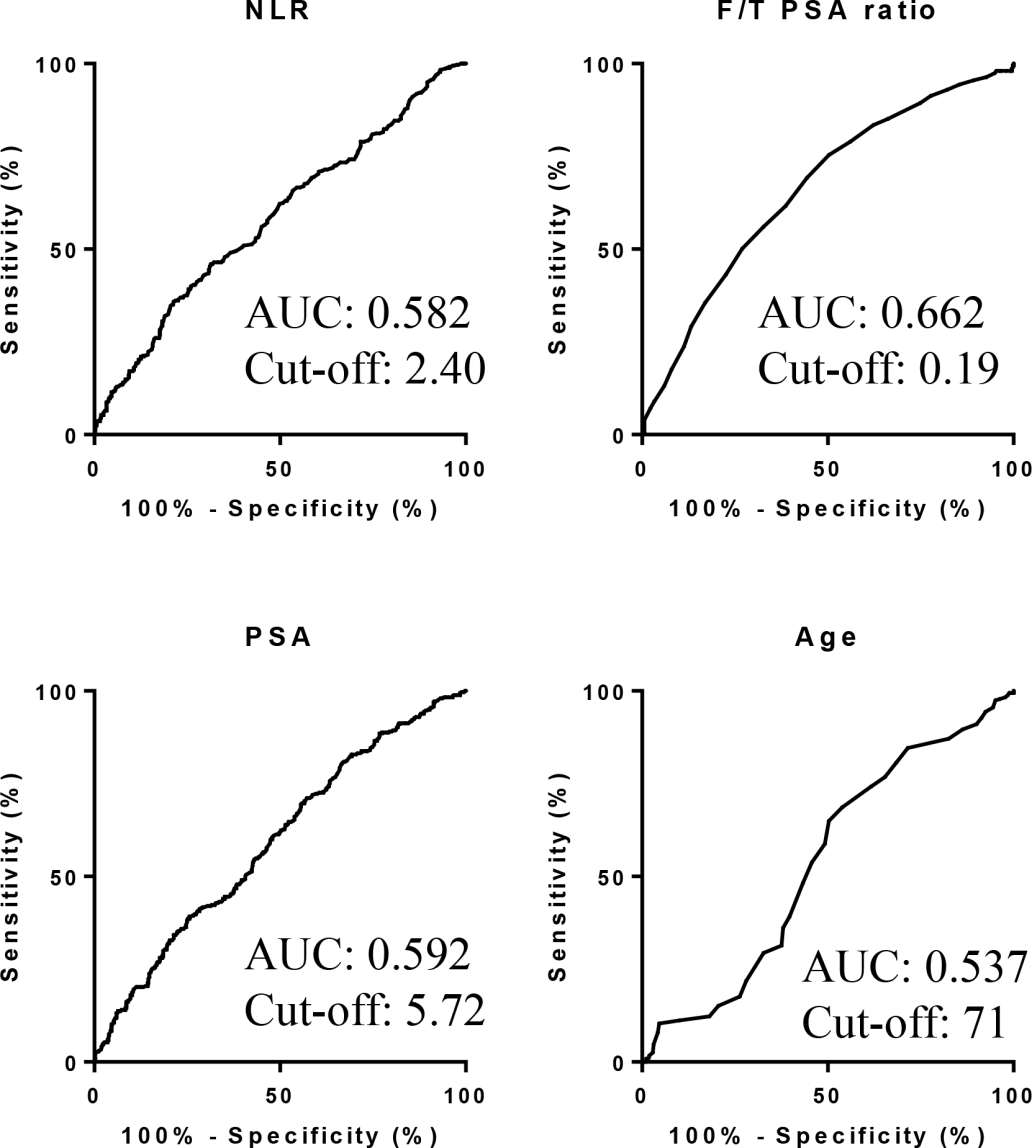
AUROC for variables to predict prostate cancer

Based on the AUROC curve, NLR cut-off point was determined as 2.40 to predict prostate cancer [Fig 5]. Multivariate analysis showed that age (*HR* = 1.91), NLR (*HR* = 2.21), PSA (*HR* = 1.54), and F/T PSA ratio (*HR* = 3.13) were independent risk factors to predict prostate cancer (Table 2). Positive and negative predictive values, using the NLR cut-off point, were 56.6% and 60.8%, respectively (Table 3). When NLR was combined with F/T PSA ratio less than 0.15, positive and negative predictive values became 80.7% and 60.1%, respectively. When F/T PSA ratios of less than 0.12 and 0.19 were used as cut-off points, in combination with NLR, positive predictive values were 83.3% and 72.9% and negative predictive values were 40.6% and 62.1%, respectively.

**Table 2:** Univariate and multivariate analyses for predicting prostate cancer

		Univariate analysis			Multivariate analysis		
	*n*	HR	95% CI	*p* value	HR	95% CI	*p* value
Age (yr)				<0.001			<0.001
<71	384	1			1		
≥71	426	1.80	1.34–2.42		1.91	1.38–2.63	
NLR				<0.001			<0.001
<2.40	228	1			1		
≥2.40	582	2.02	1.48–2.76		2.21	1.5603.13	
PSA (ng/mL)				<0.001			0.010
<5.72	297	1			1		
≥5.72	513	1.85	1.38–2.48		1.54	1.11–2.14	
F/T PSA ratio				<0.001			<0.001
<0.15	509	2.73	2.03–3.66		3.13	2.26–4.34	
≥0.15	301	1			1		

**Table 3:** Prediction of prostate cancer using NLR and F/T PSA ratio

	Sensitivity	Specificity	PPV	NPV
F/T PSA ratio <0.12	23.8% (85 of 357)	88.7% (402 of 453)	62.5% (85 of 136)	59.5 (402 of 676)
F/T PSA ratio <0.15	50.1% (179 of 357)	73.1% (331 of 453)	59.5% (179 of 301)	65.0% (331 of 509)
F/T PSA ratio <0.19	75.4% (269 of 357)	49.7% (225 of 453)	54.1% (269 of 497)	71.2% (225 of 313)
NLR ≥2.40	36.1% (129 of 357)	78.1% (354 of 453)	56.6% (129 of 228)	60.8% (354 of 582)
NLR ≥2.40 & F/T PSA ratio <0.12	9.5% (34 of 357)	98.2% (445 of 453)	83.3% (35 of 42)	40.6% (312 of 768)
NLR ≥2.40 & F/T PSA ratio <0.15	18.8% (67 of 357)	96.5% (437 of 453)	80.7% (67 of 83)	60.1% (347 of 727)
NLR ≥2.40 & F/T PSA ratio <0.19	29.4% (105 of 357)	91.4% (414 of 453)	72.9% (105 of 144)	62.1% (414 of 666)

PPV: positive predictive value, NPV: negative predictive value

## DISCUSSION

This is the first study to evaluate NLR to predict prostate cancer and reveals that higher NLR correlates with the higher incidence of prostate cancer even in men with similar PSA levels. There is increasing evidence correlating the presence of systemic inflammation with poorer cancer-specific survival in patients with several solid tumors, such as colorectal carcinoma [6, 18–23]. Moreover, nonsteroidal anti-inflammatory medications have been suggested to reduce the risk of developing prostate cancer, implying a critical correlation between inflammation and prostate carcinogenesis [18, 19]. It has previously been demonstrated that the presence of an inflammatory response can be determined by both the expression of C-reactive protein and/or an elevation in NLR [6, 24, 25]. In particular, the latter has been shown to be associated with poorer prognosis in patients with prostate cancer [15].

Although PSA has a good sensitivity, the test suffers from low specificity due to the difficulty in distinguishing patients with prostate cancer versus benign prostatic conditions [2, 26]. Other data show that men with a false positive PSA at screening are more likely to develop prostate cancer during the follow-up [27, 28]. To further improve the ability to predict prostate cancer, additional markers, including F/T PSA ratio, have been used. Despite the various cut-off levels defined in different studies, ideal cut-off values for F/T PSA ratio have not yet been determined [2, 29, 30]. Furthermore, an invasive biopsy may miss cancer in some men, given up to 20% of men will be found to have prostate cancer on a repeated biopsy [27, 31]. NLR was shown to positively correlate with elevated serum PSA [27]. The current study confirmed the findings and further showed that NLR with or without F/T PSA ratio contributed greatly to the prediction of positive and negative prostate biopsies. Accordingly, NLR either alone or in combination with F/T PSA ratio may function as a new biomarker in men who are considered to undergo prostate needle biopsy. We additionally assessed the usefulness of combinations of the factors, including age, PSA, F/T PSA ratio, and NLR. However, the sensitivities and specificities of the combinations were not superior to those of NLR or NLR + F/T PSA ratio. Based on the AUROC, the NLR cut-off value was determined as 2.40. In several studies analyzing advanced pancreatic cancer, the NLR cut-off points were around 5 [32]. In intrahepatic cholangiocarcinoma and liver metastasis from colorectal carcinoma, the NLR cut-off value was also set as 5 [27]. In renal cell carcinoma, it was 2 to 5, which varied in different studies [27].

The interactions between tumor and host immune system promote tumor cell proliferation and metastasis as well as activate the inflammatory cascade in the host, which further deteriorates the general condition of cancer patients [33]. Some studies proposed that tumor-associated neutrophils had two different states as anti-tumorigenic (N1-phenotype) and pro-tumorigenic (N2-phenotype) factors [34, 35].

Immunohistochemistry was performed to detect CD66b-positive neutrophils and CD8-positive lymphocytes in radical prostatectomy specimens (unpublished data). However, there was no statistically significant difference in the number of infiltrating CD66b-positive or CD8-positive cells between normal-appearing prostate and prostate cancer. No significant correlations between neutrophil number, lymphocyte number, or their ratio versus tumor characteristics (e.g. GS, pathological stage) or patient outcome were also seen. Further immunohistochemical analysis, especially in prostate biopsy specimens, may thus be required. There has been an immunohistochemical study in esophageal squamous cell carcinoma specimens, which demonstrated intratumoral neutrophils, CD8-positive lymphocytes, and their ratio, as seen in NLR in CBCs, correlated with disease progression [27]. However, no attempts in other tissue specimens have been made to determine the role of NLR in tumorigenesis or tumor progression as a biomarker.

There are limitations as a retrospective study. Because we extracted the data electrically, the detailed information about the criteria for performing prostate needle biopsy was unconfirmed. Therefore, there might be a bias to select patients who underwent the biopsy. Importantly, we assessed the role of NLR as a predictor of prostate cancer only in men who had a PSA value of 4–10 and underwent prostate biopsy. It is also possible that the initial prostate biopsy failed to identify existing adenocarcinoma in a subset of patients.

In conclusion, our data indicate that NLR functions as a biomarker to predict prostate cancer in men who undergo prostate needle biopsy. Combination of NLR with F/T PSA ratio likely increases both positive predictive value and negative predictive value of prostate needle biopsy.

## References

[1] Polascik TJ, Oesterling JE, Partin AW (1999). Prostate specific antigen: a decade of discovery-what we have learned and where we are going. The Journal of urology.

[2] Erol B, Gulpinar MT, Bozdogan G, Ozkanli S, Onem K, Mungan G, Bektas S, Tokgoz H, Akduman B, Mungan A (2014). The cutoff level of free/total prostate specific antigen (f/t PSA) ratios in the diagnosis of prostate cancer: a validation study on a Turkish patient population in different age categories. The Kaohsiung journal of medical sciences.

[3] Karakiewicz PI, Benayoun S, Begin LR, Duclos A, Valiquette L, McCormack M, Benard F, Saad F, Perrotte P (2007). Chronic inflammation is negatively associated with prostate cancer and high-grade prostatic intraepithelial neoplasia on needle biopsy. International journal of clinical practice.

[4] Wolters T, Roobol MJ, Schroder FH, van der Kwast TH, Roemeling S, van der Cruijsen-Koeter IW, Bangma CH, van Leenders GJ (2008). Can non-malignant biopsy features identify men at increased risk of biopsy-detectable prostate cancer at re-screening after 4 years?. BJU international.

[5] Xue P, Kanai M, Mori Y, Nishimura T, Uza N, Kodama Y, Kawaguchi Y, Takaori K, Matsumoto S, Uemoto S, Chiba T (2014). Neutrophil-to-lymphocyte ratio for predicting palliative chemotherapy outcomes in advanced pancreatic cancer patients. Cancer medicine.

[6] Gomez D, Morris-Stiff G, Toogood GJ, Lodge JP, Prasad KR (2008). Impact of systemic inflammation on outcome following resection for intrahepatic cholangiocarcinoma. Journal of surgical oncology.

[7] Chua W, Charles KA, Baracos VE, Clarke SJ (2011). Neutrophil/lymphocyte ratio predicts chemotherapy outcomes in patients with advanced colorectal cancer. British journal of cancer.

[8] Azab B, Bhatt VR, Phookan J, Murukutla S, Kohn N, Terjanian T, Widmann WD (2012). Usefulness of the neutrophil-to-lymphocyte ratio in predicting short- and long-term mortality in breast cancer patients. Annals of surgical oncology.

[9] Dalpiaz O, Pichler M, Mannweiler S, Martin Hernandez JM, Stojakovic T, Pummer K, Zigeuner R, Hutterer GC (2014). Validation of the pretreatment derived neutrophil-lymphocyte ratio as a prognostic factor in a European cohort of patients with upper tract urothelial carcinoma. British journal of cancer.

[10] Jung MR, Park YK, Jeong O, Seon JW, Ryu SY, Kim DY, Kim YJ (2011). Elevated preoperative neutrophil to lymphocyte ratio predicts poor survival following resection in late stage gastric cancer. Journal of surgical oncology.

[11] Demirtas A, Sabur V, Akinsal EC, Demirci D, Ekmekcioglu O, Gulmez I, Tatlisen A (2013). Can neutrophil-lymphocyte ratio and lymph node density be used as prognostic factors in patients undergoing radical cystectomy?. TheScientificWorldJournal.

[12] Walsh SR, Cook EJ, Goulder F, Justin TA, Keeling NJ (2005). Neutrophil-lymphocyte ratio as a prognostic factor in colorectal cancer. Journal of surgical oncology.

[13] Ohno Y, Nakashima J, Ohori M, Hatano T, Tachibana M (2010). Pretreatment neutrophil-to-lymphocyte ratio as an independent predictor of recurrence in patients with nonmetastatic renal cell carcinoma. The Journal of urology.

[14] Rosenberg L, Lawlor GO, Zenlea T, Goldsmith JD, Gifford A, Falchuk KR, Wolf JL, Cheifetz AS, Robson SC, Moss AC (2013). Predictors of endoscopic inflammation in patients with ulcerative colitis in clinical remission. Inflammatory bowel diseases.

[15] Minardi D, Scartozzi M, Montesi L, Santoni M, Burattini L, Bianconi M, Lacetera V, Milanese G, Cascinu S, Muzzonigro G (2015). Neutrophil-to-lymphocyte ratio may be associated with the outcome in patients with prostate cancer. Springerplus.

[16] van Soest RJ, Templeton AJ, Vera-Badillo FE, Mercier F, Sonpavde G, Amir E, Tombal B, Rosenthal M, Eisenberger MA, Tannock IF, de Wit R (2015). Neutrophil-to-lym phocyte ratio as a prognostic biomarker for men with metastatic castration-resistant prostate cancer receiving first-line chemotherapy: data from two randomized phase III trialsdagger. Ann Oncol.

[17] Sonpavde G, Pond GR, Armstrong AJ, Clarke SJ, Vardy JL, Templeton AJ, Wang SL, Paolini J, Chen I, Chow-Maneval E, Lechuga M, Smith MR, Michaelson MD (2014). Prognostic impact of the neutrophil-to-lymphocyte ratio in men with metastatic castration-resistant prostate cancer. Clinical genitourinary cancer.

[18] Kawahara T, Ishiguro H, Hoshino K, Teranishi J, Miyoshi Y, Kubota Y, Uemura H (2010). Analysis of NSAID-activated gene 1 expression in prostate cancer. Urologia internationalis.

[19] Ishiguro H, Kawahara T (2014). Nonsteroidal anti-inflammatory drugs and prostatic diseases. BioMed research international.

[20] Coussens LM, Werb Z (2002). Inflammation and cancer. Nature.

[21] Gunter MJ, Stolzenberg-Solomon R, Cross AJ, Leitzmann MF, Weinstein S, Wood RJ, Virtamo J, Taylor PR, Albanes D, Sinha R (2006). A prospective study of serum C-reactive protein and colorectal cancer risk in men. Cancer research.

[22] Zhang K, Kaufman RJ (2008). From endoplasmic-reticulum stress to the inflammatory response. Nature.

[23] Kawahara T, Miyoshi Y, Sekiguchi Z, Sano F, Hayashi N, Teranishi J, Misaki H, Noguchi K, Kubota Y, Uemura H (2012). Risk factors for metastatic castration-resistant prostate cancer (CRPC) predict long-term treatment with docetaxel. PloS one.

[24] Zahorec R (2001). Ratio of neutrophil to lymphocyte counts-rapid and simple parameter of systemic inflammation and stress in critically ill. Bratislavske lekarske listy.

[25] McMillan DC, Canna K, McArdle CS (2003). Systemic inflammatory response predicts survival following curative resection of colorectal cancer. The British journal of surgery.

[26] Thompson IM, Pauler DK, Goodman PJ, Tangen CM, Lucia MS, Parnes HL, Minasian LM, Ford LG, Lippman SM, Crawford ED, Crowley JJ, Coltman CA (2004). The New England journal of medicine.

[27] McDonald AC, Vira MA, Vidal AC, Gan W, Freedland SJ, Taioli E (2014). Association between systemic inflammatory markers and serum prostate-specific antigen in men without prostatic disease - the 2001–2008 National Health and Nutrition Examination Survey. The Prostate.

[28] Kilpelainen TP, Tammela TL, Roobol M, Hugosson J, Ciatto S, Nelen V, Moss S, Maattanen L, Auvinen A (2011). False-positive screening results in the European randomized study of screening for prostate cancer. European journal of cancer.

[29] De Marzo AM, Platz EA, Sutcliffe S, Xu J, Gronberg H, Drake CG, Nakai Y, Isaacs WB, Nelson WG (2007). Inflammation in prostate carcinogenesis. Nature reviews Cancer.

[30] Shafique K, Proctor MJ, McMillan DC, Qureshi K, Leung H, Morrison DS (2012). Systemic inflammation and survival of patients with prostate cancer: evidence from the Glasgow Inflammation Outcome Study. Prostate cancer and prostatic diseases.

[31] Ploussard G, Nicolaiew N, Marchand C, Terry S, Allory Y, Vacherot F, Abbou CC, Salomon L A (2013). Risk of repeat biopsy and prostate cancer detection after an initial extended negative biopsy: longitudinal follow-up from a prospective trial. BJU international.

[32] Kou T, Kanai M, Yamamoto M, Xue P, Mori Y, Kudo Y, Kurita A, Uza N, Kodama Y, Asada M, Kawaguchi M, Masui T, Mizumoto M, Yazumi S, Matsumoto S, Takaori K (2015). Prognostic model for survival based on readily available pretreatment factors in patients with advanced pancreatic cancer receiving palliative chemotherapy. Int J Clin Oncol.

[33] Mantovani A, Allavena P, Sica A, Balkwill F (2008). Cancer-related inflammation. Nature.

[34] Wang J, Jia Y, Wang N, Zhang X, Tan B, Zhang G, Cheng Y (2014). The clinical significance of tumor-infiltrating neutrophils and neutrophil-to-CD8+ lymphocyte ratio in patients with resectable esophageal squamous cell carcinoma. J Transl Med.

[35] Fridlender ZG, Sun J, Kim S, Kapoor V, Cheng G, Ling L, Worthen GS, Albelda SM (2009). Polarization of tumor-associated neutrophil phenotype by TGF-beta: ‘N1’ versus ‘N2’ TAN. Cancer Cell.

